# Multiple pregnancies: Nordic perspective and challenges

**DOI:** 10.1111/aogs.70258

**Published:** 2026-05-14

**Authors:** Vedran Stefanovic

**Affiliations:** ^1^ Department of Obstetrics and Gynecology, Fetomaternal Medical Center Helsinki University Hospital and University of Helsinki Helsinki Finland

Although multiple pregnancies are relatively rare, they are associated with substantially higher risks of adverse maternal and fetal outcomes than singleton pregnancies. These include up to a 4‐ to 5‐fold increase in perinatal mortality, largely due to preterm birth, as well as higher rates of pre‐eclampsia, gestational diabetes, cesarean delivery, and postpartum hemorrhage. In the Nordic countries, the incidence of multiple pregnancies is approximately 1.2%–1.7% of all pregnancies.^1^ After increasing from the 1980s onward because of assisted reproductive technology (ART), the multiple birth rate has stabilized or declined slightly since the early 2000s, largely owing to the adoption of single‐embryo transfer. High‐order multiple pregnancies are less common in the Nordic countries than in many other Western settings, and multifetal pregnancy reduction has also contributed to this trend. All Nordic countries provide publicly funded universal maternity care, and non‐invasive prenatal testing (NIPT) has recently become more widely available for twin pregnancies, although national practices still vary.

Monochorionic twins account for only about 30% of all twin gestations but contribute disproportionately to twin perinatal morbidity and mortality. Twin‐to‐twin transfusion syndrome (TTTS) is one of the most serious complications and remains a major cause of fetal loss before viability in advanced cases.^2^ Fetoscopic laser photocoagulation (FLP) of placental vascular anastomoses is the standard treatment for TTTS. Because TTTS is rare and treatment requires highly specialized surgical expertise, centralization of care in dedicated fetal therapy centers has been widely recommended.

The Nordic Fetal Therapy Alliance represents a collaborative model involving Denmark, Norway, Finland, and Sweden. Fetal surgery is currently provided in three referral centers: Rigshospitalet in Copenhagen, Karolinska University Hospital in Stockholm, and Helsinki University Hospital in Helsinki.^3^ Shared clinical guidelines and protocols for preoperative evaluation, treatment, and follow‐up have been developed to support consistent counseling and high‐quality postoperative care across the region. The alliance also facilitates mobility of both specialists and patients, thereby strengthening access to expertise in a field that is rapidly evolving. Since spring 2019, all Norwegian TTTS cases have been referred to centers in Denmark or Sweden for evaluation and treatment, whereas Icelandic cases have been managed elsewhere for geographical and historical reasons. In the prospective observational cohort study by Nørgaard et al.,^3^ monochorionic twin pregnancies complicated by TTTS and treated with FLP were recruited from Sweden, Norway, Denmark, and Finland between 2019 and 2022. The authors reported an overall postnatal survival rate of 74%, with at least one surviving twin in 86% of cases and survival of both twins in 64%. These outcomes are comparable to those reported by other established international fetal therapy centers. Notably, many of the included cases were managed during the COVID‐19 pandemic, when international travel was significantly restricted.

This Nordic collaboration has marked an important step forward for fetal therapy in low‐volume settings. In addition to improving clinical access and procedural centralization, it has also created opportunities for education, training, and research for the next generation of fetal therapy specialists.

Multifetal pregnancy reduction (MFPR) is a first‐trimester or early second‐trimester procedure in which the total number of fetuses is reduced by one or more in order to lower the risk of very preterm birth. It improves survival of the remaining fetus or fetuses, and reduces long‐term morbidity. Selective termination (ST), in contrast, usually refers to reduction of a fetus affected by a severe structural or genetic abnormality. The first reported ST was performed in Sweden in 1978 in a dichorionic pregnancy involving a fetus with Hurler's disease.^4^

The global use of MFPR increased sharply after the introduction of ART in the early 1980s. Unlike termination of a singleton pregnancy, MFPR and ST should be performed only in tertiary centers with advanced ultrasound expertise, access to fetal interventions, and multidisciplinary counseling involving fetal medicine specialists, clinical geneticists, neonatologists, and psychological support services. These procedures raise complex clinical and ethical questions, and management is strongly influenced by legislation, national care structures, and access to specialized services.

Recent Danish studies have added important evidence to this field.^5^, ^6^, ^7^, ^8^ Their particular strength lies in the use of nationwide registry data of high quality combined with equal access to publicly funded healthcare, comprehensive ultrasound screening, and relatively liberal legislation. These studies have also enabled assessment of long‐term outcomes in reduced and non‐reduced pregnancies.

Plurality clearly matters. In a national Danish cohort study, Rasmussen et al.^5^ examined outcomes in quadrichorionic quadriamniotic quadruplet pregnancies with and without fetal reduction. Most pregnancies were reduced to twins, whereas only a few remained non‐reduced or were reduced to singletons. Adverse pregnancy outcomes were most common in non‐reduced quadruplets and lower in pregnancies reduced to twins, although reduced quadruplets did not achieve outcomes equivalent to those of dichorionic diamniotic twin pregnancies. The findings support the clinical value of reduction in high‐order multiple pregnancies, while also underscoring the procedural risks.

Similarly, Kristensen et al.^6^ studied all trichorionic triplet pregnancies in Denmark between 2008 and 2018. Most women underwent fetal reduction to twins, and reduced pregnancies had significantly lower risks of adverse pregnancy outcomes than non‐reduced triplet pregnancies. At the same time, reduction was associated with a procedure‐related increase in miscarriage risk. In a related analysis, reduction from three fetuses to two was also associated with a substantially lower long‐term risk of severe neurodevelopmental disorders among live‐born children.^7^

In another national cohort, Kristensen reported outcomes in 9735 dichorionic twin pregnancies, of which 172 were reduced for maternal or fetal indications.^8^ Reduced twin pregnancies had a significantly lower overall risk of pregnancy complications than non‐reduced dichorionic twin pregnancies.

Finnish data provide an additional Nordic perspective. In a retrospective cohort from Helsinki University Hospital, 54 MFPR and ST cases managed between 2007 and 2019 were evaluated.^9^ Most twin pregnancies were spontaneous, whereas all quadruplet and quintuplet pregnancies, and more than half of triplet pregnancies resulted from ART initiated abroad. Most ST procedures in twin pregnancies involved an anomalous co‐twin, although substantial proportion of STs in twin pregnancies and reduction to singletons was on maternal request in otherwise normal dichorionic twin pregnancies. The overall risk of total pregnancy loss following MFPR or ST was 11.1%.

One particularly important policy issue concerns dichorionic twin pregnancies reduced on maternal request. In the Finnish cohort, this applied to a substantial proportion of cases. In Denmark, however, MFPR in twin pregnancies on maternal request was not permitted, even though termination of the entire pregnancy was legally allowed until 12 weeks.^8^ This created a striking inconsistency in practice and may have contributed to termination of otherwise healthy twin pregnancies under the legislative framework at the time when the dichorionic twins cohort was published.^8^ However, Denmark has updated its abortion law in 2025, extending the legal limit for abortion on request from 12 to 18 weeks of pregnancy.

What are the remaining challenges and future perspectives? Further reduction in the rate of multiple births should remain one of the goals in Nordic reproductive medicine. Continued commitment to single‐embryo transfer is central to this effort, as is access to MFPR for triplet and other high‐order pregnancies after appropriate counseling creating a more informed, supportive, and compassionate framework for parents facing the complexities of MFPR.^10^ A harmonized legislative framework across the Nordic countries would also help reduce inequities in access to pregnancy termination on maternal request that still exist and therefore might prevent cross‐border care seeking or so‐called abortion tourism.

Comparing the perinatal mortality rates across countries poses a challenge due to variation in the definition of legislation and different registration practices, particularly regarding pregnancies with MFPR and multiple pregnancies where one twin dies before birth.^11^

At the same time, the growing use of NIPT in twin pregnancies introduces new challenges, including no‐call results and false‐positive findings related to the vanishing twin phenomenon and placental mosaicism. These issues warrant further collaborative research. Although the Nordic countries already benefit from large, reliable twin registry resources and established research networks, important knowledge gaps remain. The Nordic Network of Fetal Medicine, which connects Nordic and Baltic countries and covers approximately 350 000 deliveries annually, would provide an excellent platform for future scientific cooperation.^12^

## Data Availability

Data sharing not applicable to this article as no datasets were generated or analysed during the current study.
